# Antimicrobial Effect and the Mechanism of Diallyl Trisulfide against *Campylobacter jejuni*

**DOI:** 10.3390/antibiotics10030246

**Published:** 2021-03-02

**Authors:** Yuanyue Tang, Fengming Li, Dan Gu, Wenyan Wang, Jinlin Huang, Xinan Jiao

**Affiliations:** 1 Key Laboratory of Prevention and Control of Biological Hazard Factors (Animal Origin) for Agri-Food Safety and Quality, Ministry of Agriculture of China, Yangzhou University, Yangzhou 225009, China; tangyy@yzu.edu.cn (Y.T.); lfm1009813208@163.com (F.L.); 006491@yzu.edu.cn (D.G.); wangwyyzu@163.com (W.W.); 2 Jiangsu Key Lab of Zoonosis/Jiangsu Co-Innovation Center for Prevention and Control of Important Animal Infectious Diseases and Zoonoses, Yangzhou University, Yangzhou 225009, China; 3 Joint International Research Laboratory of Agriculture and Agri-Product Safety, Yangzhou University, Yangzhou 225009, China

**Keywords:** *Campylobacter jejuni*, diallyl trisulfide (DATS), antimicrobial activity

## Abstract

*Campylobacter jejuni* is an important foodborne pathogen causing campylobacteriosis. It can infect humans through the consumption of contaminated chicken products or via the direct handling of animals. Diallyl trisulfide (DATS) is a trisulfide compound from garlic extracts that has a potential antimicrobial effect on foodborne pathogens. This study investigated the antimicrobial activity of DATS on *C. jejuni* by evaluating the minimal inhibitory concentrations (MICs) of *C. jejuni* 81-168, and fourteen *C. jejuni* isolates from chicken carcasses. Thirteen of 14 *C. jejuni* isolates and 81-176 had MICs ≤ 32 μg/mL, while one isolate had MIC of 64 μg/mL. Scanning electron microscopy (SEM) analysis showed the disruption and shrink of *C. jejuni* bacterial cell membrane after the DATS treatment. A time-killing analysis further showed that DATS had a dose-dependent in vitro antimicrobial effect on *C. jejuni* during the 24 h treatment period. In addition, DATS also showed an antimicrobial effect in chicken through the decrease of *C. jejuni* colony count by 1.5 log CFU/g (cloacal sample) during the seven-day DATS treatment period. The transcriptional analysis of *C. jejuni* with 16 μg/mL (0.5× MIC) showed 210 differentially expression genes (DEGs), which were mainly related to the metabolism, bacterial membrane transporter system and the secretion system. Fourteen ABC transporter-related genes responsible for bacterial cell homeostasis and oxidative stress were downregulated, indicating that DATS could decrease the bacterial ability to against environmental stress. We further constructed five ABC transporter deletion mutants according to the RNA-seq analysis, and all five mutants proved less tolerant to the DATS treatment compared to the wild type by MIC test. This study elucidated the antimicrobial activity of DATS on *C. jejuni* and suggested that DATS could be used as a potential antimicrobial compound in the feed and food industry.

## 1. Introduction

*Campylobacter* is an important foodborne pathogen causing human infections through the consumption of contaminated meat products. Campylobacteriosis has been known as the most frequently reported zoonotic disease in humans in the world during the previous decade [1]. *Campylobacter* spp. has caused 96 million human infection cases, which is the second most important foodborne pathogen for human infections [2]. The infection of *Campylobacter* is mainly caused by direct contact with animals during meat handling and the consumption of uncooked poultry products [3]. In addition, the frequent occurrence of multidrug-resistant *Campylobacter* pathogenic has increased the difficulties for the infection treatment. In China, *C. jejuni* has been reported to be increased resistance to quinolones and tetracyclines from 1994 to 2010, which increased the difficulty of treating this bacteria by antibiotics [4]. Since *Campylobacter* spp. are commensal bacteria colonizing in the poultry digestive system, the antimicrobial strategies to reduce *Campylobacter* at the farm level are considered as the priority [3,5]. Thus, the investigation of intervention strategies and antimicrobial alternatives that do not further increase bacterial resistance is important for preventing the transmission of *Campylobacter* in poultry production.

Garlic (*Allim sativum*) is a common plant used for its health benefits since ancient times. Several scientific and clinical studies have been carried out to investigate the biological activity of compounds that are included in garlic essential oil and provide benefits to immunity, exert antioxidant and antibacterial activities, and protect against infection and inflammation [6,7,8,9]. The garlic bulb contains high amounts of γ-glutamylcysteine, which could be oxidized to an inactive derivate-alliin, and further metabolized to an active derivate-allicin (diallyl thiosulfinate) by alliinase releasing during chopping or cutting [10,11]. Allicin could rapidly disintegrate into sulfur-rich compounds, including diallyl sulfide (DAS), diallyl disulfide (DADS) and diallyl trisulfide (DATS) [12]. These diallyl sulfide derivatives are mainly attributed the antimicrobial activities of the garlic essential oil, and the numbers of sulfur atoms determine their antimicrobial capability [13,14]. These organosulfur compounds are considered as active ingredients originated from plants that are responsible for the inhibition of bacterial growth [15].

DATS is a trisulfide compound with a molecular weight of 162 g/mol. Compared to DAS and DADS, DATS has a greater number of sulfur atoms, which explains its stronger antimicrobial activity [16,17]. A previous study showed that the sulfides from virulence genes could regulate the quorum sensing (QS) signal molecules and extracellular virulence factors of *Pseudomonas aeruginosa* both in vivo and in vitro [18]. Further studies also proved that DADS could inhibit the expression of *las*, *rhl* and *pqs* for QS autoinducer synthesis [19]. In addition, diallyl sulfide derivatives from garlic also proved to inhibit the QS transcriptional regulators LuxR and LasR [20,21]. In addition, DATS has been shown to inhibit the production of *Microcystis aeruginosa* biomass by impairing the photosynthetic center PSII and alteration of APA in previous research [22].

Since *Campylobacter* can only multiply in the chicken intestinal tract, interventional strategies at the farm level to reduce *Campylobacter* are preferable [3]. This study aimed to explore the antimicrobial effect of DATS on *C. jejuni* and investigate the underlying mechanism of its action, which could provide more evidence on the potential use of DATS as a natural and efficient antimicrobial compound against *C. jejuni* in the feed and food industry.

## 2. Results

### 2.1. Antimicrobial Effect of DATS on Campylobacter spp.

**Figure 1:**
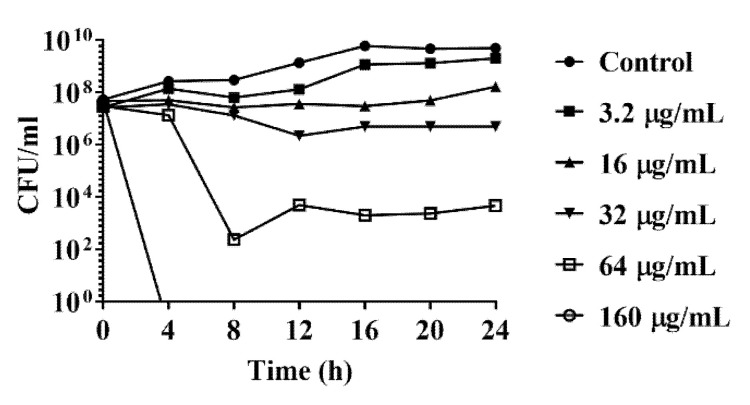
Time-kill curve of *C. jejuni* strain 81-176 with DATS. The treatment concentration of DATS included 3.2 μg/mL, 16 μg/mL, 32 μg/mL, 64 μg/mL and 160 μg/mL. 2% of dimethyl sulfoxide without DATS were used as a negative control. The data represented the means ± standard deviation.

The antimicrobial activity of DATS was evaluated by MICs over a serial of concentration on strain 81-176 and 15 strains from chicken samples collected during a previous study [23] (Table 1). The MIC value of DATS to strain 81-176 was 32 μg/mL, while MICs for chicken isolates were ranged from 1 to 64 μg/mL (Table 1). 0.5% DMSO was used as a negative control since it did not have an effect on the growth of *C. jejuni* (Figure 1).

A time-kill analysis was further conducted to evaluate the DATS antimicrobial activity against *C. jejuni*. The time-kill analysis of *C. jejuni* strain 81-176 was conducted with DATS with DATS at a dose-dependent manner, including 3.2 μg/mL (0.1× MIC), 16 μg/mL (0.5× MIC), 32 μg/mL (1× MIC), 64 μg/mL (2× MIC), 160 μg/mL (5× MIC). *C. jejuni* treated with 3.2 μg/mL DATS was slightly eliminated until 16 h, while *C. jejuni* treated with 16 and 32 μg/mL of DATS were decreased from 10^8^ to 10^7^ CFU/mL after 24 h (Figure 1). When the concentration of DATS increased to 64 μg/mL, the concentration of *C. jejuni* was decreased to 10^2^ CFU/mL (Figure 1). The bacteria were fully eliminated when treated with 160 μg/mL DATS (Figure 1). These results indicated that the DATS could act as an antimicrobial compound to eliminate the growth of *C. jejuni*.

### 2.2. Morphological Changes Caused by DATS

**Figure 2:**
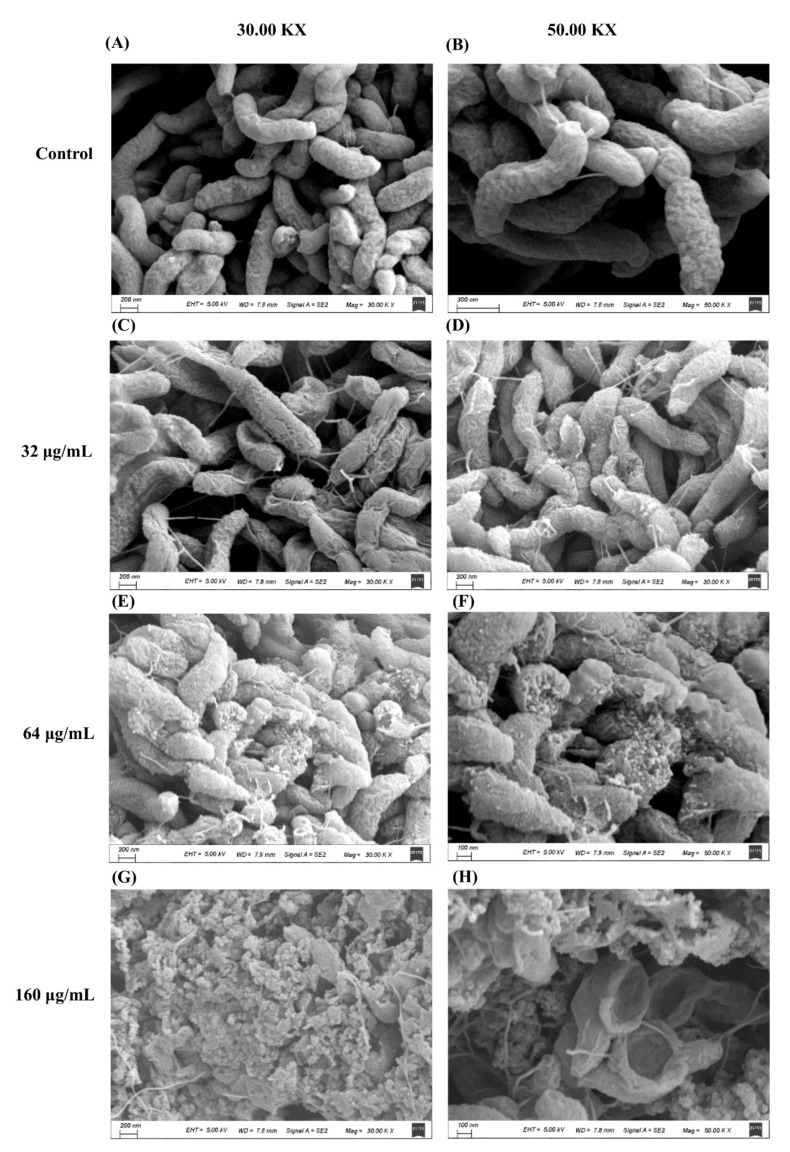
Scanning electron microscopy photography of *C. jejuni* strain 81-176 planktonic cell under DATS treatment and the control after 24 h. The control was *C. jejuni* culture without DATS treatment (**A**,**B**). The treatment concentrations of DATS included 32 μg/mL (**C**,**D**), 64 μg/mL (**E**,**F**) and 160 μg/mL (**G**,**H**).

Scanning electron microscopy (SEM) was used to investigate the morphological changes of *C. jejuni* after exposure to DATS (32 μg/mL, 64 μg/mL, 160 μg/mL). The cells of the control sample showed a helical shape and complete cell structure (Figure 2A,B). After treatment with DATS, the cell structure of *C. jejuni* showed various changes (Figure 2C–H). The cell structure was deformed, and the cell membrane was damaged after treatment with 32 μg/mL of DATS (Figure 2C,D), while the bacterial cells were shrunk and disrupted after treatment with 64 μg/mL DATS (Figure 2E,F). The treatment of 160 μg/mL DATS could disintegrate the bacterial cell and form voids on the cell surface (Figure 2G,H). The SEM results indicated that DATS could induce remarkable morphological changes in *C. jejuni*, including cell deformation and shrink cell membrane damage and cell lysis at high treatment concentration.

### 2.3. Antimicrobial Effect of DATS In Vivo

**Figure 3:**
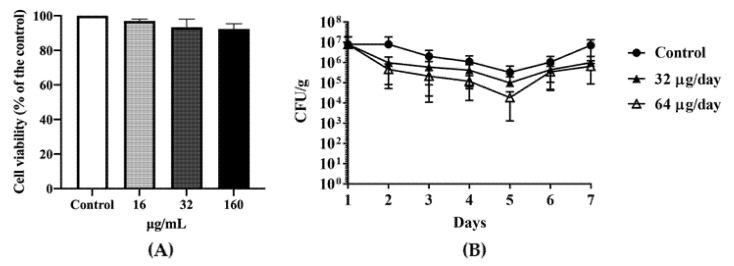
The in vivo antimicrobial effect of DATS. (**A**) the MTT((3-(4,5-Dimethylthiazol-2-yl)-2,5-diphenyltetrazolium bromide)) assay of Hela cell under a serial of DATS treatments. (**B**) *C. jejuni* 81-176 colonization by colony-forming unit (CFU) per gram of cloacal samples in Hy-line variety white chickens during seven-day DATS treatment. The dark circle represented the control group treated with PBS; the dark up-pointing triangle represented the group treated with 32 μg DATS per day; the white up-pointing triangle represented the group treated with 64 μg DATS per day. Each group contained five chickens. CFU data indicated an average of three animals with a standard deviation.

An MTT(3-(4,5-Dimethylthiazol-2-yl)-2,5-diphenyltetrazolium bromide) cell viability assay indicated that DATS did not significantly affect cell viability (Figure 3A), which at the 16 μg/mL (0.5× MIC), about 98% of cells were viable, while at the 32 μg/mL (1× MIC) and 160 μg/mL (5× MIC) about 95% of cells were still viable. Depends on the results of MICs and MTT assay, we divided the chickens into two groups with orally feeding 1 mL of 32 μg/mL (1× MIC) DATS and 1 mL of 64 μg/mL DATS (2× MIC) per day for seven days (21-day age to 28-day age), respectively. On day 21, the *Campylobacter* cell number of the cloacal samples ranged from 4 × 10^5^ CFU/g to 2 × 10^7^ CFU/g before DATS treatment. During the seven-day DATS treatment, both DATS-treated groups showed a decrease in *Campylobacter* colonization (Figure 3B) until day 5, which the 32 μg DATS group decreased 1 logCFU/g and the 64 μg DATS-treated group decreased approximately 1.5 logCFU/g, respectively. However, both treated groups showed an increase in *Campylobacter* cell numbers on day 6, while *Campylobacter* cell numbers increased to 1 × 10^7^ CFU/g of the cloacal samples on day 7 (Figure 3B).

### 2.4. Transcriptomic Analysis

**Figure 4:**
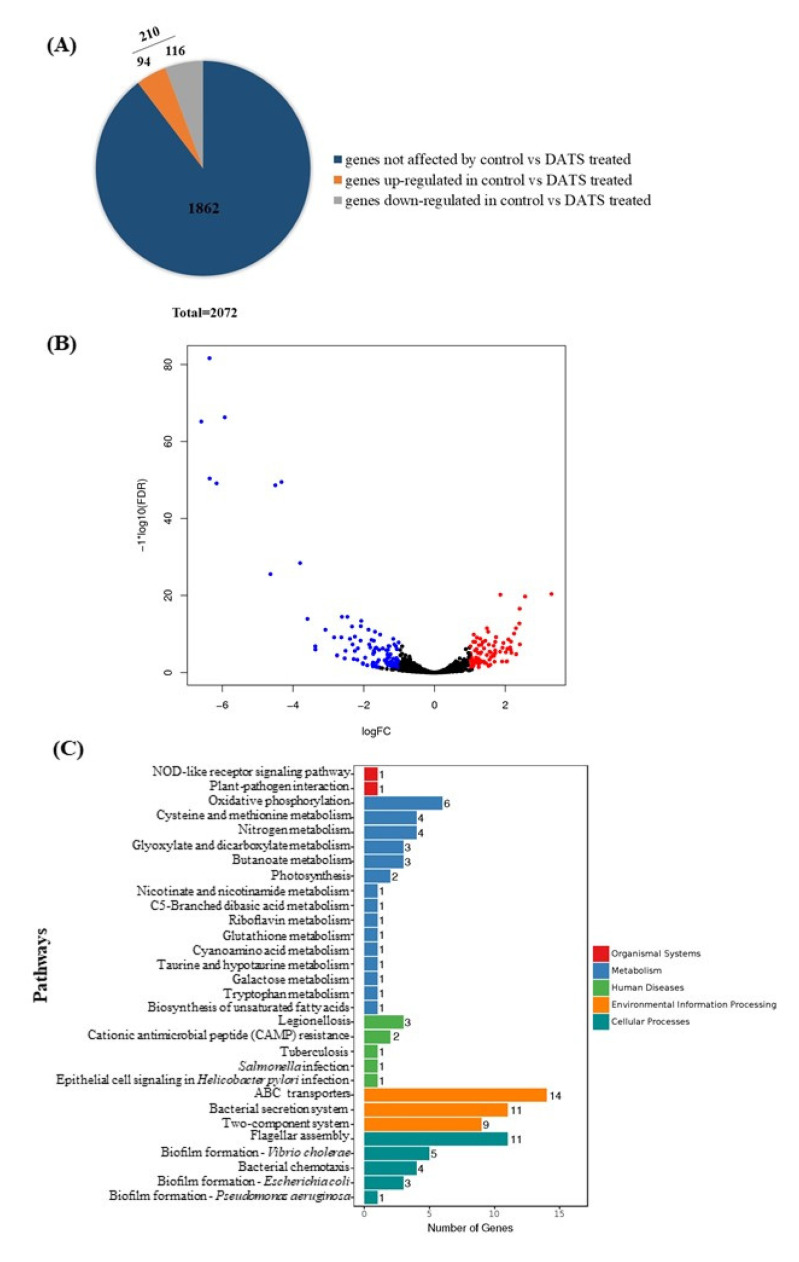
Transcriptional analysis of *C. jejuni* strain 81-176 under 16 μg/mL DATS treatment. (**A**) The distribution of total differentially expression genes (DEGs), which blue color represented genes not affected by DATS; orange color represented upregulated genes under DATS treatment; ad gray color represented downregulated genes under DATS treatment. (**B**) The volcano plot for global comparison of transcription profiles between DATS treatment and the control. Red spots represented upregulated genes under DATS treatment; blue spots represented downregulated genes under DATS treatment. (**C**) Statistical enrichment of DEGs in the KEGG pathway.

To explore the potential antimicrobial mechanisms of DATS against *C. jejuni*, RNA-seq was carried out to analyze gene expression profile changes of *C. jejuni* 81-176 as a response to the DATS treatment. In total, *C. jejuni* 81-176 contained 2072 genes (Figure 4A). The RNA-seq data indicated that 210 genes were significantly differentially expressed (*p* < 0.05) after treatment with 16 μg/mL (0.5× MIC) of DATS, of which 116 genes were upregulated, and 94 genes were downregulated (Figure 4A, Table S3). The volcano plot of the RNA-seq analysis was generated to visualize the differentially expressed genes treated with 16 μg/mL DATS (Figure 4B).

The Kyoto Encyclopedia of Genes and Genomes (KEGG) pathway enrichment analysis was used to unravel the biological functions of differentially expressed genes (DEGs) at molecular and cellular levels (Figure 4C). DEGs involved in the DATS treatments were divided into the pathways, including the transmembrane transporter (*n* = 14), the flagellar assembly (*n* = 11) the bacterial secretion system (*n* = 11), and the nitrogen metabolism (*n* = 4) and the biofilm formation (*n* = 3) (Table S3). Interestingly, we found 14 of the ABC transporter proteins were downregulated, which may be involved in the DATS antimicrobial activity.

### 2.5. The Effect of DATS on the Transmembrane Transporter

**Figure 5:**
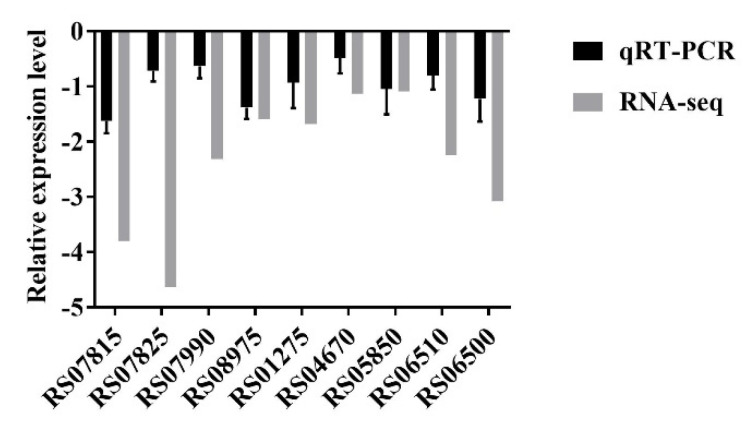
Validation of RNA-seq data by quantitative real-time PCR. The data represented the means ± standard deviation.

An RNA-seq analysis indicated that transmembrane transporter-related genes were significantly downregulated after treatment with 16 µg/mL DATS. Therefore, nine significantly downregulated transmembrane transport genes (Table 2) were selected to verify our findings by qRT–PCR and these results were consistent with the RNA-seq data (Figure 5). The effect of five transmembrane transporter-related genes was further evaluated by constructing the deletion mutant of *C. jejuni* 81-176 and determining MICs of these mutants (Table 3). Our results showed that MICs of all *C. jejuni* mutants for DATS were decreased at a range from 8 to 16 µg/mL, while the MIC of the wild type was 32 µg/mL (Table 3), indicating that transmembrane transporter could be an important factor the elimination of *C. jejuni* growth by DATS.

## 3. Discussion

*C. jejuni* is one of the most important foodborne pathogens causing a growing global public concern, and it is transmitted by the consumption of contaminated poultry products [24]. This study investigated the antimicrobial activity of DATS against *C. jejuni* and the antibacterial mechanism of DATS on the transcriptome level. DATS has been known as an organosulfur precursor from allicin. Similar to all other thiosulfinates from allium extracts, DATS could transform into alkeney alkene thiosulfinate allicin by alliinase after crashing the alliin [25]. The antimicrobial activities of allium extracts against pathogens, such as *Escherichia coli* O157, *Bacillus anthracis*, *Streptococcus iniae*, *Candida albicans*, and *Enterococcus faecalis,* have been previously reported [11]. DATS is one of the most important antimicrobial compounds in the *Allium* essential oil, which have been reported to exert antimicrobial activity against pathogens, including *Staphylococcus aureus*, *Staphylococcus epidermidis* and *Micrococcus lysodeikticus* [26]. In addition, the application of garlic extracts as a preservative in food products is widely encouraged in meat products, which could also contribute to the flavor during the cooking process [11]. Thus, the application of natural antimicrobial compounds is advantageous for the perceived quality apart from the improvement of safe food and feed [27].

Poultry products are the main vector for *C. jejuni* contamination. This study evaluated the antimicrobial activities of DATS on 14 of *C. jejuni* isolates from chicken carcasses in a slaughterhouse as previously reported [23]. The MIC experiments revealed that nine of 14 *C. jejuni* isolates could be eliminated by DATS with a concentration ≤ of 8 μg/mL, four chicken isolates and the reference strain 81-176 had a MIC value of 32 μg/mL, and one isolates had a MIC value of 64 μg/mL (Table 1). The time-kill experiments of strain 81-176 revealed that the antimicrobial effect of DATS over time was dose-dependent (Figure 1). The bacterial loads were decreased with the increase of DATS treatment concentration over the 24 h treatment period in vitro. The further SEM analysis also showed the loss of flagellar with the presence of DATS (Figure 5). Moreover, in the MIC experiment, we conducted the MTT assay by Hela cell and the animal experiment to evaluate the effect of DATS in vivo. Our results showed that DATS did not show the cytotoxic effect on the growth of Hela cells (Figure 3A). Moreover, our in vivo experiment showed that DATS could decrease approximately 2 logCFU/g of *Campylobacter* in cloacal samples in vivo during the first five days of feeding, but the effect was increased afterward, indicating *Campylobacter* might develop a resistance system during the continuously treating with the DATS. The presence of acidity of the stomach might also influence the bioactivity of DATS [25]. Further investigation could be necessary to assess the resistance and survival of *C. jejuni* with the presence of DATS. Garlic extracts have shown antimicrobial activity by decreasing the virulence of *Salmonella* spp., *E. coli* and *P. aeruginosa* [28]. The antimicrobial effects of DATS or other diallyl sulfide derivatives have not been studied in *C. jejuni* before. On the other hand, several studies have been conducted on investigating the antimicrobial effect of DAS, DADS, DAT, DATS on *P. aeruginosa*; DATS showed an equal antimicrobial effect to the multidrug-resistant *P. aeruginosa* as the garlic essential oil, and both were stronger compared to DAS, DADS and DAT according to the MIC results [19,21]. A previous study treated *P. aeruginosa* by 2.84 mg/L for 120 h and observed a 50% decrease of cell density by the hemocytometer counting method, while the inhibition efficiency was increased with the increasing of treatment dose [22].

By KEGG pathway enrichment analysis with the RNA-seq data, DEGs were grouped to the metabolism pathways, the environmental information pathways, and cellular processes pathways (Figure 4C). The downregulated pathway in ABC transporters was interesting, which were selected for further analysis. Fourteen genes were enriched in the ABC transporter pathway, which was mainly involved in the periplasmic space and plasma membrane (Table S3). The ABC transporter is the multidomain complex membrane responsible for transferring molecules, such as lipids, steroids, and toxins, to cross the cellular membranes using the hydrolysis of ATP [29]. The TonB transporter system is located on the bacterial outer membrane, which is responsible for binding and transporting ferric chelates [30]. The downregulation of TobB system-related genes and the ABC transporter would decrease the activity of reducing ferric ion (Fe^3+^) to ferrous ion (Fe^2+^); the excess Fe^2+^ could induce the formation of free radicals and harm the cell [30]. The zinc importer ZupT is known to play an important role in bacterial homeostasis, which protects bacteria to grow in metal devoid environments against oxidative stress and helps bacteria colonize in the host [31]. The downregulated genes in the transporter system on the cell membrane indicated that DATS treatment could decrease the bacterial ability to keep the homeostasis and the ability to against oxidative stress, which were also consistent with our SEM results that *C. jejuni* cells were shrunk and disrupted after DATS treatment (Figure 2). The effect of DATS on the ABC transporter was further evaluated by constructing the five deletion mutants. All mutants showed decreased MIC values, which further proved that the decreasing activity of the ABC transporter could be involved in the antibacterial activity of DATS. The transcriptional profiles indicate that DATS act as an antimicrobial compound by decrease the activity of the transporter system. Ajoene is another sulfur-containing compound from garlic extract with antimicrobial activities, which could inhibit the thiol–enzyme by interacting with thiol groups [32]. However, the mechanism of DATS interacts with the pathogen remained unclear under the transcriptional level analysis, which required further investigation.

## 4. Materials and Methods

### 4.1. Bacterial Strains and Culture Methods

All *C. jejuni* strains used in this study are shown in Table 1. Plasmids and competent cells used in this study are shown in Table S1. All *C. jejuni* strains were cultured on Mueller–Hinton (MH) agar (CM0337, Oxoid Limited, Hampshire, UK) plates supplemented with 5% fiber-free sheep blood at 42 °C for 24 h under a microaerobic condition (85% N_2_, 10% CO_2_, 5% O_2_).

### 4.2. Minimum Inhibitory Concentration (MIC) Analysis

The minimum inhibitory concentrations (MICs) of DATS were determined by broth micro-dilution, as recommended by the Clinical and Laboratory Standards Institute (CLSI) guidelines [33]. DATS (SMB00289, Sigma-Aldrich, St. Louis, MO, USA) was dissolved in dimethyl sulfoxide (DMSO) with a concentration of 20 mg/mL as a stock solution. Briefly, two-fold serial dilutions of DATS were prepared in Mueller–Hinton broth (MHB) (CM0405, Oxoid Limited, Hampshire, UK) and added 100 µL per well in 96-well microtiter plates, then 100 µL of *C. jejuni* bacterial suspension was added to each well. The final bacterial concentration was 5 × 10^5^ CFU/mL with a serial concentration of DATS from 2 µg/mL to 1024 µg/mL with two-fold dilution. The 2% DMSO was used as a negative control. The plates were incubated at 42 °C for 24 h under microaerobic conditions. MICs were determined as the lowest concentration of DATS that eliminated the visible growth of *C. jejuni* strains.

### 4.3. Time Kill Analysis

*C. jejuni* were inoculated on MH agar plate with 5% sheep blood and incubated for 24 h at 42 °C under microaerobic conditions. The overnight cultured *C. jejuni* were suspended in MHB and adjust to an OD_600_ value of 0.05 with the bacterial cell concentration of 5 × 10^7^ CFU/mL. Serial dilutions of DATS were added in *C. jejuni* broth culture with the final concentrations of 3.2 μg/mL, 16 μg/mL, 32 μg /mL, 64 μg/mL, and 160 μg/mL. *C. jejuni* broth culture without DATS treatment was used as the control. All *C. jejuni* broth cultures were incubated at 42 °C, 180 rpm, under microaerobic conditions. The concentration of bacteria was determined by plate counts with serial dilution every four hours until 24 h.

### 4.4. Scanning Electron Microscopy

Scanning electron microscopy (SEM) was performed to investigate the morphological changes of *C. jejuni* after the treatment of DATS as described in the previous study with some modifications [24]. *C. jejuni* 81-176 was inoculated in MHB overnight at 42 °C and diluted to 1 × 10^9^ CFU/mL before adding a serial concentration of DATS (32 μg/mL, 64 μg/mL and 160 μg/mL), and incubated for 24 h at 42 °C under microaerobic condition. *C. jejuni* 81-176 in MHB without DATS was used as a negative control. The DATS-treated samples and the negative control were washed three times with sterilized water and centrifugation at 8000× *g* for 10 min at 4 °C. Bacterial pellets were fixed with 2.5% (*w/v*) glutaraldehyde at 4 °C for 12 h. The bacterial were rinsed three times with 0.1 M phosphate buffer, dehydrated in graded ethanol series (25%, 50%, 70%, 80%, 90%) for 10 min. Samples were freeze-dried and coated in layer gold and examined by GeminiSEM 300 (Carl Zeiss, Oberkochen, Germany).

### 4.5. MTT Cytotoxicity Assay of DATS

The cell viability was evaluated by the MMT cell viability assay in Hela cell. Cells were cultured in 10 mL DMEM with 10% of fetal bovine serum and 100 U/mL penicillin and 100 μg/mL streptomycin and cultured at the 37 °C under 5% CO_2_ with humidity. Confluent cell cultures were trypsinized and inoculated in 96-well cell culture plates with a concentration of 1 × 10^5^ cells per well and incubated at 37 °C under 5% CO_2_ with humidity incubator for 24 h. Cell medium was replaced with serum-free medium containing different concentrations of DATS (16 μg/mL, 32 μg/mL, 160 μg/mL) and incubated at 3 7 °C under 5% CO_2_ for 24 h. Cells incubated in serum-free medium without DATS treatment were the negative control. The MTT cell viability assay was conducted according to the previous study with some modifications [34]. In brief, 20 μL MTT was added to PBS with a concentration of 5 mg/mL and incubated for 3 h. MTT were discarded 100 μL formazan. The quantification of living cells was evaluated by the absorbance of each well were measured under 570 nm wavelength. The viability of cells was calculated by considering the control as 100% of cell viability. All experiments were carried out with triplicates.

### 4.6. Animal Experiments

One-day-old Hy-Line White chickens for this study were purchased from Jinan Sipaffrey Poultry Co., Ltd. (Jinan, China) and confirmed to be *Campylobacter* free by biological test. The cloacal swabs were obtained from each chicken on day five for the confirmation of *Campylobacter* free, which each swab sample was inoculated in the MH broth with 5% fiber-free sheep blood at 42 °C for 24 h under a microaerobic condition, and further inoculated on mCCDA plates and incubated for 24 h under the same condition. Chickens were orally inoculated with 10^7^ CFU of 81-176 to ensure the 100% *Campylobacter* colonized chicken before DATS treatment [35]. Five chickens were grouped in each separate rearing isolator and supplied with commercial feed and water. *Campylobacter* in each chicken was quantified by cloacal samples before DATS treatment on day 21, in which cloacal samples were collected by 10 μL loop, suspended in 1 mL PBS and quantified by serial dilution on mCCDA plates. Each chicken group was orally treated with 1 mL of different concentrations of DATS (16 μg/mL, 32 μg/mL, and 160 μg/mL) per day until day 28. On each day, *Campylobacter* in each chicken was quantified by the cloacal sample as described above.

### 4.7. RNA-seq Analysis

*C. jejuni* 81-176 was inoculated in MHB with or without 0.5× MIC of DATS (16 μg/mL) and incubated under microaerobic conditions for 24 h at 42 °C, 180 rpm. The bacteria pellets were collected by centrifugation at 8000× *g* for 5 min at 4 °C. The total RNA was extracted by RNeasy plus mini kit (Qiagen, Hilden, Germany). The three parallel RNA samples were sequenced by the illumine HiSeq (GENEWIZ, Suzhou, China). Quality control and data filtering were processed by Cutadapt (version 1.9.1), and then the filtered data were aligned to the reference genome via software Bowtie 2 (v2.2.6). HTSeq (v0.6.1p1) was used to estimate gene expression levels from the clean date. The different expression analysis was conducted by the DESeq 2 Bioconductor package and adjusted by Benjamini and Hochberg’s approach for controlling the false discovery rate [36]. Padj of genes was set <0.05 to determine differentially expressed transcripts.

### 4.8. Quantitative Real-Time Polymerase Chain Reaction (qRT–PCR)

*C. jejuni* 81-176 was cultured with or without 0.5× MIC of DATS (16 μg/mL), and the total RNA was extracted using an RNeasy plus mini kit (Qiagen, Hilden, Germany). DNase I was used to eliminate the gDNA contamination. The first single-strand cDNA was synthesized from 1 μg of total RNA by RT reagent kit (TaKaRa, Dalian, China) with random hexamers as a primer in a 20 μL reaction mixture. qRT–PCR was carried out in ABI PRISM 7500 real-time PCR system (Applied Biosystems, Foster City, CA, USA) using a FastStart Universal SYBR Green Master (Roche, Mannheim, Germany), and the specific primers are shown in Table S2. The relative quantity of the mRNA was calculated using the ΔCt method. The 16S rRNA gene was used as endogenous control, which was equally transcribed in both treated and untreated bacterial cells. The raw sequence data of RNA-seq have been deposited in the European Nucleotide Archive database with the accession number PRJEB42739.

### 4.9. Construction of C. jejuni Mutant Strain

The deletion mutant strains were constructed as previously described [37]. Genes selected for the deletion mutants were related to the transmembrane transport, which was significantly downregulated during the DATS treatment, including RS07815, RS07825, RS07990, RS06500, and RS05850 (Table 2). Each deletion mutant was obtained by replacing the targeted gene with kanamycin-resistant cassette aphA-3. In brief, the up and down flanking regions of the targeted and kanamycin-resistant cassette from pRY107 were amplified by PCR with primers list in Table S2. PCR fragments and pMD-20T vector were ligated together by a one-step cloning kit (Vazyme, Nanjing, China). The recombination plasmid was electroporated into *C. jejuni* 81-176 and selected on CCDA agar containing 50 mg/mL kanamycin and 50 mg/mL ampicillin. All deletion mutant strain was verified by sequencing.

## 5. Conclusions

DATS is known as a compound naturally occurring in garlic extracts. This study demonstrated that DATS has antimicrobial activity against *C. jejuni*. The mechanisms of the antimicrobial action were evaluated at the phenotypical and transcription level. We observed that DATS could destruct the bacterial cell membrane and decrease the activity of the bacterial membrane transporter system. Moreover, we also observed a decrease in *C. jejuni* cell number after DATS treatment in vivo. Our results indicated that DATS could be used as an alternative natural compound against *C. jejuni* in meat products. Further study should be carried out to elucidate the whole mechanism of DATS antimicrobial activities against pathogens.

## Figures and Tables

**Table 1:** Bacteria strains and minimal inhibitory concentrations of diallyl trisulfide (DATS) in the present study.

Strains	Host	MICs (μg/mL)	Source
81-176	Human	32	National Collection of Type Culture
YZU01384	Chicken	64	[23]
YZU01388	Chicken	8	[23]
YZU01391	Chicken	32	[23]
YZU01394	Chicken	8	[23]
YZU01364	Chicken	1	[23]
YZU01367	Chicken	32	[23]
YZU01372	Chicken	32	[23]
YZU01375	Chicken	8	[23]
YZU01378	Chicken	32	[23]
YZU01381	Chicken	4	[23]
YZU01385	Chicken	4	[23]
YZU01389	Chicken	2	[23]
YZU01368	Chicken	1	[23]
YZU01371	Chicken	8	[23]

**Table 2:** Transcription intensity changes of open reading frames of *C. jejuni* 81-176 treated with 16 µg/mL DATS.

Gene_ID	Fold Change	*p* Value	Biological Function
CJJ81176_RS07815	−3.799	1.98 × 10^−31^	TonB-system energizer ExbB
CJJ81176_RS07825	−4.637	1.71 × 10^−28^	Energy transducer TonB
CJJ81176_RS07990	−2.315	1.77 × 10^−9^	ABC transporter ATP-binding protein
CJJ81176_RS08975	−1.600	9.165 × 10^−6^	Major facilitator superfamily transporter
CJJ81176_RS01275	−1.681	4.55 × 10^−13^	Zinc transporter ZupT
CJJ81176_RS04670	−1.134	5.1 × 10^−4^	Membrane protein insertion efficiency factor YidD
CJJ81176_RS05850	−1.091	5.94 × 10^−9^	F0F1 ATP synthase subunit A
CJJ81176_RS06510	−2.251	1.61 × 10^−7^	ABC transporter ATP-binding protein
CJJ81176_RS06500	−3.083	1.31 × 10^−13^	ABC transporter permease

**Table 3:** MICs of *C. jejuni* 81-176 mutants in DATS.

Strain	MIC (μg/mL)
81-176	32
81-176∆*RS07815*	16
81-176∆*RS07825*	16
81-176∆*RS07990*	8
81-176∆*RS05850*	16
81-176∆*RS06500*	16

## Data Availability

The datasets for this manuscript are not publicly available because the results of this manuscript have not been published yet. Requests to access the datasets should be directed to Yuanyue Tang, tangyy@yzu.edu.cn.

## Supplementary Materials

The following are available online at https://www.mdpi.com/2079-6382/10/3/246/s1, Table S1: Strains and plasmids used in this study; Table S2: Primers used in this study; Table S3: Genes differentially expressed between DATS-treated condition and the untreated condition.
